# Hemorrhagic Cystitis Due to Cephalexin and Review of the Literature

**DOI:** 10.1177/00099228231175473

**Published:** 2023-05-19

**Authors:** Arnold Gan, Luis Delgado, Kimberly Swafford, Dayton Brown, Maulin Soneji

**Affiliations:** 1 University of Riverside School of Medicine, Riverside, CA, USA; 2 College of Osteopathic Medicine of the Pacific, Western University of Health Sciences, Pomona, CA, USA; 3 Loma Linda University School of Medicine, Loma Linda, CA, USA; 4 Division of Pediatric Infectious Diseases, Department of Pediatrics, Loma Linda University School of Medicine, Loma Linda, CA, USA

**Educational Objectives**

- Antibiottic associated hemorrhagic cystitis should be included in a broad differential for hematuria.
- Discontinuation of the offending agent can rapidly improve symptoms.

## Introduction

Hemorrhagic cystitis (HC) is a disease of the urinary bladder characterized as widespread inflammation resulting in hemorrhage from the bladder mucosa. The risk factors for HC include infection, medication use, pelvic radiation therapy, and systemic disease. For acute HC, bacterial infection is the most common infectious etiology, whereas chronic or recurrent HC is more commonly due to pelvic radiotherapy and chemotherapeutic agents.^1^ Of the chemotherapeutic agents, oxazaphosphorine alkylating agents, such as ifosfamide and cyclophosphamide, have been most implicated in the development of HC due to generation of acrolein, a urotoxic metabolite that concentrates in the bladder.^2-4^

The clinical presentation of HC ranges from microscopic hematuria with symptoms of bladder irritation to gross hematuria, passage of clots, and life-threatening bladder hemorrhage.^5^ The diagnostic criteria of HC include urinalysis (UA) findings of hematuria (>5 red blood cells (RBCs)/high power field [hpf]), adequate platelet count (>50 000/mm^3^), and negative urine culture; however, for severe or questionable cases cystoscopy or computed tomography (CT) urogram can be used for further diagnostic evaluation.^6,7^

In this report, we describe a case where gross hematuria and abdominal pain developed in a patient after oral cephalexin as outpatient treatment for osteomyelitis of the left knee.

## Case Report

A previously healthy 9-year-old male presented to the Riverside University Health System Emergency Department for swelling of his left knee associated with fever, decreased appetite, and fatigue. Lab results showed normal white blood cell (WBC) count, elevated C-reactive protein (CRP) of 8.07 mg/dL, and normal procalcitonin. Blood cultures were drawn, and synovial aspiration was performed. Joint fluid results were inconsistent with septic arthritis, and the patient was discharged home. Blood culture grew *Staphylococcus aureus*. The patient was asked to return for treatment. Left knee magnetic resonance imaging (MRI) showed changes consistent with osteomyelitis of the proximal tibia and blood cultures grew *Staphylococcus aureus* again. The patient was diagnosed with methicillin-sensitive *Staphylococcus aureus* bacteremia and osteomyelitis. He was given a 6-week course of antibiotics that began with intravenous cefazolin. After a negative blood culture at 72 hours and a CRP level <1 mg/dL, the patient was discharged with 1500 mg cephalexin orally every 8 hours.

Two days after discharge, the patient experienced sudden onset of abdominal pain associated with episodes of hematuria and presented back to the emergency department (ED). Urinanalysis showed hematuria with 333 RBCs, 7 WBCs, trace protein, and specific gravity of 1.010. One hyaline cast was found. No urine eosinophils or peripheral eosinophilia were present. Complete metabolic panel showed creatinine to be 0.7 mg/dL. Abdominal CT and ultrasound of kidney and bladder showed no signs of a renal stone. A clinical diagnosis of HC was made after ruling out other causes of hematuria such as nephrolithiasis or nephritis. Cephalexin was changed to IV cefazolin to complete the remaining 5 weeks of the original 6-week course. Subsequent lab testing showed stable creatinine and UA after 48 hours of discontinuation of cephalexin showed 5 RBCs. A peripherally inserted central catheter (PICC) line was placed, and the patient was discharged home with follow-up scheduled with infectious disease. During outpatient follow-up, hematuria did not recur.

## Discussion

While chemotherapeutic agents have most commonly been implicated in the development of drug-induced HC, cases of HC have been reported by various classes of drugs in both pediatric and adult patients. Table 1 summarizes key clinical characteristics of numerous reports that causally link antibiotics to induction of HC.

**Table 1 .** Cases of Medication Induced Hemorrhagic Cystitis.

Article	Age (years) and gender	Medication	Time until HC	Work up to confirm HC	Treatment	Antibiotic change	Days until resolution of hematuria
Relling and Schunk^8^	12/Male	Ticarcillin	7 days	UA, UC	Discontinuation of Ticarcillin	None	2 days
Marx and Alpert^9^	4/Female	Ticarcillin	10 days	UA	Discontinuation of ticarcillin	Trimethoprim-sulfamethoxazole, dicloxacillin	7 days
Marx and Alpert^9^	6/Female	Carbenicillin	9 days	UA, IV pyelogram, cystourethrogram	Discontinuation of carbenicillin	None	7 days
Joy^10^	23/Male	Piperacillin	1 day	UA	Discontinuation of piperacillin	None	Unknown
Godin et al^11^	24/Male	Methicillin	13 days	CBC, CMP, UA, bone marrow aspiration, renal biopsy	Discontinuation of Methicillin	Pristinamycin	5 days
Kim et al^12^	63/Female	Penicillin G	24 days	UA, UC, CRP	Discontinuation of penicillin G	none	8 days
Cook et al^13^	64/Male	Benzylpenicillin	11 days	UA, UC, cystoscopy and biopsy	Discontinuation of penicillin G	Vancomycin	5 days
Adlam et al^14^	72/Male	Penicillin G	28 days	UA, UC, cystoscopy and biopsy	Discontinuation of penicillin G	None	2 days
Toma et al^15^	49/Male	Penicillin G	16 days	UA, UC, CT scan	Discontinuation of penicillin G	Linezolid, aztreonam	21 days
Zeng et al^16^	37/Male	Voriconazole	27 days	RUS	Reduced dosage	None	Unknown
Pournasiri et al^17^	2//Male	Cephalexin	5 hours	UA, UC, CBC	Symptomatic care	None	1 day
Verma et al^18^	25/Male	Cephalexin	8 days	UA, UC, CBC, retrograde pyelogram, cystoscopy	Discontinuation of cephalexin	None	5 days
Verma et al^18^	24/Male	Cephalexin	9 days	UA, UC, CBC	Discontinuation of cephalexin	None	7 days
Present case	9/Male	Cephalexin	7 days	UA, UC, CBC, RUS, abdominal CT	Discontinuation of cephalexin	Cefazolin	2 days

Abbreviations: HC, Hemorrhagic cystitis; CBC, complete blood count; CMP, comprehensive metabolic panel; UA, urinalysis; UC, urine culture; IV, intravenous; CRP, C reactive protein; CT, computed tomography; RUS, renal ultrasound.

Relling and Schunk^8^ reported a 12-year-old with cystic fibrosis hospitalized for pulmonary disease exacerbation who developed gross hematuria and painful urination following ticarcillin IV and netilmicin IV treatment. Symptoms resolved 48 hours following medication withdrawal.

Kim et al reported HC in a 63-year-old female with relapsed bone and joint infection due to *Streptococcus agalactiae* of the hip joint. She was treated with penicillin G and subsequently developed urinary frequency with dysuria and hematuria on the 24th day of treatment. Symptoms resolved within 8 days of discontinuing Penicillin G.^12^ Adlam et al,^14^ Cook et al,^13^ and Toma et al^15^ described similar occurrences of HC in their patients after administration of Penicillin G.^13-15^

Zeng et al described a case of HC in a 37-year-old male diagnosed with central nervous system aspergillosis who had been treated with voriconazole. Following treatment, he developed severe HC and bladder rupture which required surgical repair.^16^ The dose of voriconazole was decreased and eventually bladder hemorrhage resolved.

Joy reports a case where a 23-year-old otherwise healthy male who participated in a study where he completed a regiment of piperacillin sodium with lignocaine 1.0 g 3 times a day for 3 days.^10^ The patient experienced microscopic hematuria 1 day after completion on UA, which showed 100 RBCs/hpf. Patient was otherwise asymptomatic. Follow-up UA 3 weeks later was normal.

Marx and Alpert^9^ reports of 2 cases where both patients were girls hospitalized for exacerbations of pulmonary disease secondary to cystic fibrosis. In the first case, the 4-year-old girl developed pyuria, microscopic hematuria, and proteinuria with urinary frequency and dysuria on the fifth day of treatment with ticarcillin and tobramycin sulfate. The antibiotic regimen was discontinued 2 days later with symptoms resolving over the next 7 days. She subsequently received ticarcillin for a pulmonary exacerbation and then piperacillin with a third exacerbation. During both of these exacerbations, she developed similar symptoms of HC. In the second case, the 6-year-old girl developed dysuria, increased urinary frequency, and microscopic hematuria on the fifth day of hospitalization after administration of IV gentamicin and carbenicillin. The microscopic hematuria progressed to gross hematuria with passage of blood clots. The antibiotic regimen was discontinued 8 days later. Urinary symptoms resolved 7 days after discontinuation of IV gentamicin and carbenicillin.

Godin et al^11^ describes a case of HC in a 24-year-old male treated with 8 grams of IV methicillin and 160 mg IM gentamicin for sternoclavicular staphylococcal arthritis. Isolated macroscopic hematuria was noted on the 13th day of treatment with increased urinary frequency and dysuria appearing a couple days later. Renal failure was observed on the 21st day with 20.3 mmol/L of urea and 0.292 mmol/L of creatine. Methicillin was replaced with pristinamycin and gentamicin dose was reduced. A generalized rash, neutropenia, and eosinophilia was subsequently observed with bone marrow aspiration confirming drug-induced agranulocytosis. Five days after methicillin discontinuation, neutropenia and urinary symptoms resolved, whereas urinary function returned to normal within 17 days.

Drug-induced HC is a rare complication, especially with antibiotic treatment. Cephalosporin and particularly cephalexin-induced HC has been reported rarely, leading us to share this pediatric case of cephalexin induced HC. To the best of our knowledge, there are only three previous reports of cephalexin-induced HC.^17,18^ Clinicians with a high suspicion of HC should consider medications as a causative agent as this can lead to early diagnosis, withdrawal of the medication, and prompt recovery.

Our patient did not demonstrate adverse effects when switched to cefazolin. The exact mechanism of cephalosporin-induced HC is not currently known, but we believe the difference in chemical structure between both first-generation agents may have played a role in our patient’s clinic presentation. Cephalexin’s molecular formula is C16H17N3O4S, and it contains a methyl and beta-(2R)-2-amino-2-phenylacetamido groups at the 3- and 7- of the cephem skeleton.^19^ It has a relatively short half life of 49.5 minutes and is 90% excreted by the urine within 6 hours.^19^ Cefazolin’s molecular formula is C14H14N8O4S3, and it contains [(5-methyl-1,3,4-thiadiazol-2-yl)sulfanyl] methyl and (1H-tetrazol-1-ylacetyl) amino side-groups at positions 3 and 7.^20^ It has a longer half life and excretion time than cephalexin with a half life of 1.8 hours and 60% urinary excretion within 6 hours.^20^ The topographical polar surface area of cephalexin is 138 angstroms squared, whereas for cefazolin it is 235 angstroms squared.^19,20^ The decreased polarity and shorter excretion time of cephalexin may contribute to its ability to induce HC over cefazolin.

In the case with our patient, he was being treated for a non-life-threatening osteomyelitis and was responding well to cefazolin. Previous instances of antibiotic induced HC usually abated once the offending agent was discontinued, as stated above. If this type of reaction were to happen again in another patient with a similar presentation, it would be advised to discontinue the offending antibiotic and switch the patient to cefazolin or nafcillin/oxacillin for the treatment of a non-life-threatening osteomyelitis.^21^

## Conclusion

We present a case of a 9-year-old male who presented to the ED for left knee swelling and fever that was diagnosed with *Staphylococcus aureus* bacteremia and osteomyelitis. He was treated and discharged with oral cephalexin, and later returned to the ED after 2 days with abdominal pain and hematuria that was diagnosed as drug induced HC. This case explains the rarity of HC being induced by antibiotics, as most cases of drug induced HC are caused by chemotherapeutic agents. We have described several cases of HC caused by antibiotics, but currently there have not been significantly documented cases of HC caused by cephalexin. This highlights the importance of keeping antibiotics on the list of possibilities when diagnosing a drug-induced HC.

## Author Contributions

AG contributed to conception and design, analysis, drafted the manuscript, critically revised the manuscript, gave final approval, and agrees to be accountable for all aspects of work ensuring integrity and accuracy. LD contributed to conception and design, analysis, drafted the manuscript, critically revised the manuscript, gave final approval, and agrees to be accountable for all aspects of work ensuring integrity and accuracy. KS contributed to conception and design, analysis, drafted the manuscript, gave final approval, and agrees to be accountable for all aspects of work ensuring integrity and accuracy. DB contributed to conception and design, analysis, drafted the manuscript, gave final approval, and agrees to be accountable for all aspects of work ensuring integrity and accuracy. MS contributed to conception and design, analysis, drafted the manuscript, critically revised the manuscript, gave final approval, and agrees to be accountable for all aspects of work ensuring integrity and accuracy.
